# Estimation of metabolite networks with regard to a specific covariable: applications to plant and human data

**DOI:** 10.1007/s11306-017-1263-2

**Published:** 2017-09-22

**Authors:** Georgios Bartzis, Joris Deelen, Julio Maia, Wilco Ligterink, Henk W. M. Hilhorst, Jeanine-J. Houwing-Duistermaat, Fred van Eeuwijk, Hae-Won Uh

**Affiliations:** 1 Department of Medical Statistics and Bioinformatics, Leiden University Medical Center, Einthovenweg 20, 2300 RC Leiden, The Netherlands; 2 Department of Biological Mechanisms of Ageing, Max Planck Institute for Biology of Aging, Joseph-Stelzmann-Strasse 9b, 50931 Cologne, Germany; 3 São Paulo State University, FCA/UNESP, Botucatu, SP CEP 18610-307 Brazil; 4 Wageningen Seed Lab, Laboratory of Plant Physiology, Wageningen University, Droevendaalsesteeg 1, 6708 PB Wageningen, The Netherlands; 5 Department of Statistics, School of Mathematics, University of Leeds, Leeds, LS2 9JT UK; 6 Biometris, Wageningen University, P.O. Box 16, 6700 AC Wageningen, The Netherlands

**Keywords:** Network reconstruction, Incorporating relevant information, Metabolites, Study design

## Abstract

**Introduction:**

In systems biology, where a main goal is acquiring knowledge of biological systems, one of the challenges is inferring biochemical interactions from different molecular entities such as metabolites. In this area, the metabolome possesses a unique place for reflecting “true exposure” by being sensitive to variation coming from genetics, time, and environmental stimuli. While influenced by many different reactions, often the research interest needs to be focused on variation coming from a certain source, i.e. a certain covariable $\mathbf {X}_m$.

**Objective:**

Here, we use network analysis methods to recover a set of metabolite relationships, by finding metabolites sharing a similar relation to $\mathbf {X}_m$. Metabolite values are based on information coming from individuals’ $\mathbf {X}_m$ status which might interact with other covariables.

**Methods:**

Alternative to using the original metabolite values, the total information is decomposed by utilizing a linear regression model and the part relevant to $\mathbf {X}_m$ is further used. For two datasets, two different network estimation methods are considered. The first is weighted gene co-expression network analysis based on correlation coefficients. The second method is graphical LASSO based on partial correlations.

**Results:**

We observed that when using the parts related to the specific covariable of interest, resulting estimated networks display higher interconnectedness. Additionally, several groups of biologically associated metabolites (very large density lipoproteins, lipoproteins, etc.) were identified in the human data example.

**Conclusions:**

This work demonstrates how information on the study design can be incorporated to estimate metabolite networks. As a result, sets of interconnected metabolites can be clustered together with respect to their relation to a covariable of interest.

## Introduction

**Fig. 1:**
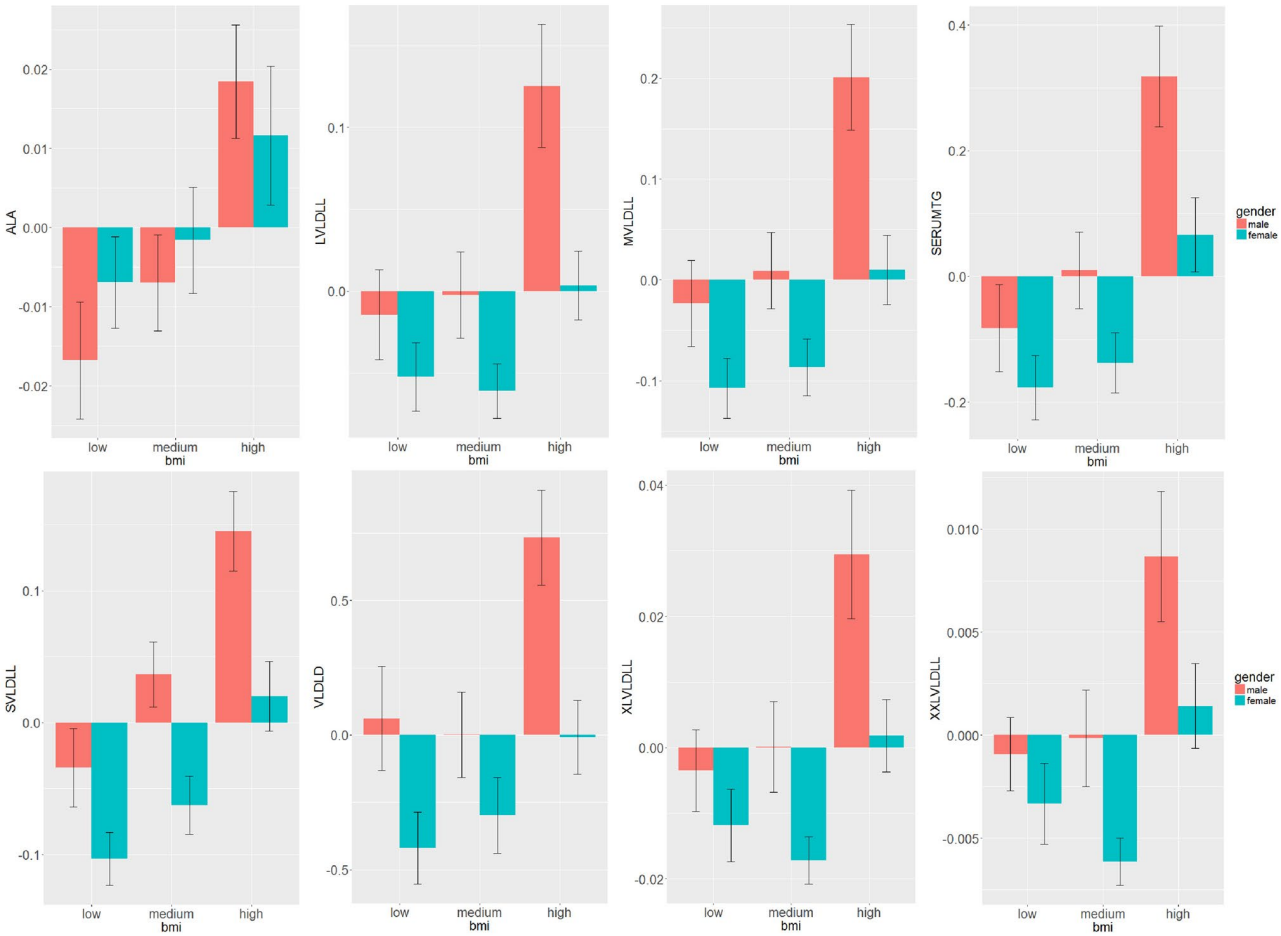
Barplots representing the metabolite concentrations in humans by BMI class and sex for metabolites belonging in the VLDL module

In recent years, network analysis of biological datasets has become an increasingly popular tool for studying the relationships between large numbers of variables that occur in omics research on transcripts, metabolites, proteins and others. In networks, variables are represented by nodes and their relationships, direct and indirect interactions (physical or functional), are represented by edges or links. One is often interested in the joint distribution of a set of variables conditional on a particular covariable. For example, one may want to study the relations between a set of metabolites with regard to body mass index (BMI). As an illustration, in Fig. 1 we show the concentrations of eight very large density lipoprotein (VLDL) particles that are associated with BMI and gender in a similar way, i.e. males have higher VLDL concentrations than women; low VLDL concentrations are associated to low and medium BMI categories, and high VLDL concentrations to high BMI. The aim of the current paper is to detect these groups of metabolites with similar relationships by using network analysis.

For network estimation, networks with different features are typically used for representing networks, i.e. undirected or directed networks. In undirected networks, edges connecting two nodes do not have a direction indicating a symmetric relationship between them. In biology, undirected network estimation methods based on correlation are often preferred. These methods perform well with large numbers of samples and variables. However, little is known about their performance in a small sample setting. Therefore, estimating, describing, visualizing and comparing networks for relatively small samples is an ongoing topic of research (Kolaczyk and Krivitsky 2015). We will consider two methods of estimating undirected networks. The first is weighted gene co-expression network analysis (WGCNA) based on correlation (Zhang and Horvath 2005; Langfelder and Horvath 2008; Zhao et al. 2010). While WGCNA was mainly developed for analysis of gene-expression data, applications on metabolite data have been reported as well (DiLeo et al. 2011; Zhang et al. 2013). WGCNA is based on the concept of scale free networks implying the existence of a few highly connected nodes (hubs) participating in a very large number of metabolic reactions (Zhao et al. 2010). In WGCNA, the strength of the connection between nodes is typically dictated by a similarity measure (Zhao et al. 2010). The second method is the graphical LASSO (GL) (Friedman et al. 2001, 2008) based on partial correlations. For this Gaussian graphical model (GGM) based method, for two nodes not sharing a direct edge in a network the implication is that they are conditionally independent given all other variables. To obtain a sparse network an L1 penalty can be used. The penalty can be determined using a stability selection algorithm (StARS) (Liu et al. 2010) to select a stable set of edges.

For describing and comparing the estimated networks using the two methods above, we first characterize the topology of the networks by measuring three types of network concepts Dong and Horvath (2007), Horvath and Dong (2008), i.e. density, centralization, and heterogeneity.

We will consider two different data applications for estimating metabolite networks. In the first application, the metabolites are coming from an experiment with seeds in which the desiccation tolerance of these seeds was investigated as a function of genotype and a managed environment condition, or treatment. Seeds from two genotypes of the well-known plant *Arabidopsis thaliana*, the genotype Columbia-0 (Col-0) and the abscisic acid deficient mutant 2–1 (*aba2–1*) were selected. Germinated seeds at radicle protrusion were selected and either frozen in liquid nitrogen and stored at −80 °C directly or after 3d of incubation in −2.5 MPa polyethylene glycol (PEG), 5 μM ABA (ABA) or a combination of −2.5 MPa PEG + 1 μM ABA (PEG + ABA). Therefore, four treatments for metabolic profiling have been considered here: (i) no treatment (control), (ii) PEG, (iii) ABA or (iv) both PEG and ABA. In this paper, we focus on estimating metabolite networks based on genotype-related information.

The second application concerns serum metabolites of unrelated individuals coming from the capital region of Finland. In this observational study our specific interest lies in estimating networks of metabolites, conditioned on the individuals’ BMI status which might interact with individuals’ sex and age.

The rest of the paper is organized as follows. In Sect. 2, we propose our method for selecting information relevant to a certain covariable prior to network estimation. Additionally we review some existing network estimation methods. In Sect. 3, we demonstrate our network estimation approaches on metabolite data coming from plants and humans, and we conclude with a Discussion in Sect. 4.

## Methods

A network consists of a set of nodes (or vertices) connected by a set of edges (or links). In this paper we consider undirected networks of metabolites, where the nodes correspond to the metabolites, and the edges between metabolites represent their relationship. For a network of *P* nodes, the network structure can be represented by the $P\times P$ adjacency matrix $\mathbf {A}$. For undirected networks, the elements $a_{ij}$ of the adjacency matrix are defined as follows:

$$\begin{aligned} a_{ij} = {\left\{ \begin{array}{ll} 1, &{} \text {if there is an edge between node }j\text { and node }i\\ 0, &{} \text {otherwise.} \end{array}\right. } \end{aligned}$$

Note that the adjacency matrix $\mathbf {A}$ is symmetric and has zeros on the diagonal. The degree or connectivity $k_i$ of a node *i* is defined as $k_i=\sum _{j\ne i}a_{ij}$: i.e. $k_i$ equals the number of neighbors of node *i* in the network. In addition to the adjacency matrix we typically consider a $P\times P$ intensity matrix $\mathbf {W}$ where the elements $w_{ij}$ represent the strength of the relationship between node *i* and *j*. If nodes *i* and *j* are not linked, the weight $w_{ij}$ is equal to zero. Popular choices for the weights $w_{ij}$ are Pearson’s correlation coefficient, mutual information, Euclidean distance, partial correlation and topological overlap.

We will consider absolute Pearson’s correlation coefficient and partial correlations with no self-edges ($w_{ii}=0$). Analogously to the degree of a node $i\left( {k_{i} } \right),$ the strength or weighted degree is defined as $s_i=\sum _{j\ne i}w_{ij}$. The strength of a node takes into account both the connectivity as well as the weights of the edges.

### Identifying the specific parts of metabolic concentrations relevant to a covariable

The weights represent the relationships between the metabolite concentrations. In addition one might be interested in the relationships between specific parts of the metabolite concentrations: for example the part which is related to a covariable of interest. The idea is that metabolites with similar relationships with this covariable will tend to be close to each other in the network (see for example Fig. 1). The parts of the metabolite concentration concerning this specific covariable can be obtained by fitting linear regression models (or ANOVA) to the metabolic variables. Let ${\mathbf{Y}} = \left( {{\mathbf{Y}}^{{(1)}} , \ldots ,{\mathbf{Y}}^{{(P)}} } \right)$ be the concentrations of *P* metabolites and let $\mathbf {Y}^{(p)}$ be the vector of concentrations for the *p*th metabolite. Assume that $\mathbf {Y}$ follows a multivariate Gaussian distribution $\mathbf {Y} \sim N_P (\varvec{\mu },\varvec{\Sigma })$. From *m* covariables let $\mathbf {X}_m$ be the categorical covariable of main interest. The remaining $m-1$ covariables are denoted as ${\mathbf{X}}^{{( - m)}} = \left\{ {{\mathbf{X}}_{1} ,{\mathbf{X}}_{2} , \ldots ,{\mathbf{X}}_{{m - 1}} } \right\}$.

Now we propose the following regression model:

1 $$\begin{aligned} \mathbf {Y}^{(p)}=\beta ^{(p)}_{0}+\varvec{\beta }_1^{(p)}\mathbf {X}_{m}+\sum _{\varvec{\delta }\in \varvec{\varDelta }}\varvec{\gamma }^{(p)}_{\varvec{\delta }}\prod _{j=1}^{m-1}\mathbf {X}_{j}^{\varvec{\delta }_j}+\sum _{\varvec{\delta }\in \varvec{\varDelta }}\varvec{\eta }^{(p)}_{\varvec{\delta }} \mathbf {X}_{m}\circ \prod _{j=1}^{m-1}\mathbf {X}_{j}^{\varvec{\delta }_j}+\varvec{\varepsilon }^{(p)}, \end{aligned}$$

where $\varepsilon ^{p} \sim N\left( {0,\sigma _{{(p)}}^{2} } \right)$ represents the random noise, $\varvec{\varDelta }$ is the space with elements all vectors of length $m-1$ with all combinations of zeros and ones, except all zeros, i.e. $\varvec{\varDelta }=\{ (1,0,\ldots ,0),(0,1,\ldots ,0),\ldots ,(1,1,\ldots ,1)\}$, and $\circ$ is the Hadamard product. The term $\sum _{\varvec{\delta }\in \varvec{\varDelta }}\varvec{\gamma }_{\varvec{\delta }}\prod _{j=1}^{m-1}\mathbf {X}_{j}^{\varvec{\delta }_j}$ models all main effects and second and higher order interaction terms of covariables in $\mathbf {X}^{(-m)}$. For example, for $m-1=2$, $\sum _{\varvec{\delta }\in \varvec{\varDelta }}\varvec{\gamma }_{\varvec{\delta }}\prod _{j=1}^{2}\mathbf {X}_{j}^{\varvec{\delta }_j}=\varvec{\gamma }_{10}\mathbf {X}_{1}+\varvec{\gamma }_{01}\mathbf {X}_{2}+\varvec{\gamma }_{11}\mathbf {X}_{1}\circ \mathbf {X}_{2}$. Similarly, the term $\sum _{\varvec{\delta }\in \varvec{\varDelta }}\varvec{\eta }_{\varvec{\delta }} \mathbf {X}_{m}\circ \prod _{j=1}^{m-1}\mathbf {X}_{j}^{\varvec{\delta }_j}$ models all terms interacting with $\mathbf {X}_m$. To ensure identifiability of all parameters a level of the covariable is used as reference category.

The relevant information for the *p*th metabolite with regard to the categorical covariable $\mathbf {X}_m$ is given by:

2 $$\begin{aligned} \hat{\mathbf {Y}}^{(p)}=\hat{\varvec{\beta }}_1^{(p)}\mathbf {X}_{m}+\sum _{\varvec{\delta }\in \varvec{\varDelta }}\hat{\varvec{\eta }}^{(p)}_{\varvec{\delta }} \mathbf {X}_{m}\circ \prod _{j=1}^{m-1}\mathbf {X}_{j}^{\varvec{\delta }_j}. \end{aligned}$$

Now the edge between node *p* and *q* in a network can be based on correlation between $\hat{\mathbf{Y }}^{(p)}$ and $\hat{\mathbf{Y }}^{(q)}$.

### Network estimation methods

We consider two network approaches, namely WGCNA based on pairwise correlations between metabolites and GL based on partial correlations.

#### Weighted gene co-expression network analysis (WGCNA)

WGCNA (Horvath and Dong 2008; Zhang and Horvath 2005; Langfelder and Horvath 2008) has been developed to efficiently analyze the correlation patterns among genes using gene expression data from microarray experiments. Network construction with WGCNA is typically followed by identifying clusters (modules) of highly correlated genes.

*Estimation of networks—modeling the intensity matrix* Complex networks may display non-trivial topological features, such as a heavy tail in the empirical distribution of the degree of the nodes (the number of edges connected to a node). In biology, often networks with many low degree nodes and a few high degree nodes are of interest. Such networks are called scale free. To determine whether a network is scale free the log of the degree frequency ($\log (P(k))$) is plotted against the logarithm of the degree ($\log (k)$). A linear relationship indicates that the network is scale-free. A scale-free degree distribution can be expressed as $P(k) \propto k^\gamma$ and in a weighted case, i.e. in the context of WGCNA, the intensity $w_{ij}$ can be written on a logarithmic scale, $\log w_{{ij}} = \gamma \log \left| {{\text{cor}}\left( {y_{i} ,y_{j} } \right)} \right|$ for $\gamma>1$. The threshold parameter $\gamma$ might be chosen in a way such that the network approximately satisfies the scale-free topology criterion.

In our experience, however, not all biological datasets yield scale free networks. If the network is not scale free $\gamma$ will be determined by the amount of noise in the dataset. For two Gaussian random variables, the magnitude of random noise for correlation coefficients from *N* samples is $1/\sqrt{N}$. In order to sufficiently suppress low correlations due to noise, we take the smallest value for $\gamma$ in such a way that the total noise is smaller than one (personal communication with Peter Langfelder):

3 $$\begin{aligned} \dfrac{P(P-1)}{2(\sqrt{N})^{\gamma }}<1. \end{aligned}$$

*Module identification* A network might consist of a set of modules of closely interconnected metabolites. Average linkage hierarchical clustering based on a dissimilarity measure is a popular method to define a dendrogram of the network. The modules are obtained by cutting this tree (the two-step dynamic hybrid algorithm) (Langfelder et al. 2008). Here, we will use the following dissimilarity measure:

4 $$Diss\left( {w_{{ij}} } \right) = 1 - \left| {w_{{ij}} } \right|.$$

#### Graphical LASSO (GL)

GL is another popular approach to obtain a network for a set of variables. Assume that the metabolite concentrations (*N* by *P* data matrix $\mathbf {Y}$) follow a multivariate Gaussian distribution with mean vector $\varvec{\mu }$ and variance-covariance matrix $\varvec{\Sigma }$. For simplicity we can assume that the data are centered, i.e. $\varvec{\mu }=\mathbf {0}$. The inverse covariance matrix $\varvec{\Sigma }^{-1}$ is the precision matrix. When its elements are equal to zero, the pair of metabolites is conditionally independent given the other metabolites. A Gaussian graphical model (Lauritzen 1996) is a network based on these conditional independence relationships.

To estimate $\varvec{\Sigma }^{-1}$ a penalized log-likelihood approach can be used. Define the precision matrix $\varvec{\Theta }=\varvec{\Sigma }^{-1}$. Under the Gaussian model, the log-likelihood function is given by (up to a constant):

5 $$\ell (\mathbf \Theta )\sim \log |\varvec{\Theta } |-\text{ tr }(\mathbf {S}\varvec{\Theta }),$$

where $\mathbf {S}=\mathbf {Y}^\top \mathbf {Y} /N$ is the sample covariance matrix. Maximizing expression 5 with respect to $\varvec{\Theta }$ leads to the maximum likelihood estimate $\hat{\varvec{\Theta }}$. Note that the elements of $\hat{\varvec{\Theta }}$ are in general not exactly equal to zero. Further in the high-dimensional setting ($P>N$), $\mathbf {S}$ is singular and cannot be inverted to obtain $\hat{\varvec{\Theta }}$.

Therefore a penalized version of the log likelihood is typically maximized (Friedman et al. 2008; Hastie et al. 2009; Friedman et al. 2001; Rothman et al. 2008; Yuan and Lin 2006). For the Lasso penalty the log likelihood function is as follows:

6 $$\begin{aligned} {} \ell _\lambda (\varvec{\Theta })\sim \log |\varvec{\Theta } |-\text{ tr }(\mathbf {S}\varvec{\Theta })-\lambda ||\varvec{\Theta }||_1, \end{aligned}$$

where $\lambda$ is a non-negative tuning parameter. For $\lambda =0$, the resulting network will be fully connected. While $\lambda$ increases, sparsity is induced to the estimated $\hat{\varvec{\Theta }}$ and the network starts to lose edges to the point that no more edges are left. Consequently, elements of the resulting estimated precision matrix will be exactly equal to zero.

The tuning parameter $\lambda$ can be chosen so that the number of edges are biologically relevant and straightforward to interpret. A statistical approach for choosing $\lambda$ can be based on Akaike’s information criterion (AIC), Bayesian information criterion (BIC), or *K*-fold cross-validation. To obtain a stable edge set with a low false discovery rate, stability approach for regularization selection (StARS) (Liu et al. 2010) is an attractive approach. It provides a penalty corresponding to the least amount of regularization that simultaneously makes a network sparse and is replicable under random sampling. GL with StARS is implemented in the *huge* R package (Zhao et al. 2012).

### Network evaluation

We now consider several measures to describe a specific network or a subset of a network and to compare networks, namely density, centralization and heterogeneity (Dong and Horvath 2007). Let $\mathbf {M}$ be the weights of the edges of the nodes in a network, i.e. $\mathbf {M}$ is the $P \times P$ matrix $\mathbf {W}$ for WGCNA after suppressing low correlations due to noise or the stability matrix estimated by StARS for GL.

The network density of $\mathbf {M}$ is the mean of these weights and is estimated by:

7 $${\text{Density}}\,({\mathbf{M}}) = \sum\limits_{i} {\sum\limits_{{j \ne i}} {\frac{{w_{{ij}} }}{{P(P - 1)}}} } = \frac{{\bar{s}}}{{(P - 1)}},$$

where $\bar{s}$ is the mean of *s*. A value close to one indicates high interconnectedness.

The centralization is the difference between the strength of the most connected node in the network with respect to the average network and is given by

8 $$\begin{aligned} \begin{aligned} \text{Centralization}\,(\mathbf {M})&= \dfrac{P}{P-2}\left( \dfrac{\text{ max }(s)}{P-1}-{\text{density}} \right) \\&=\dfrac{P}{(P-1)(P-2)}\left(\text{max }(s)-\bar{s} \right) \\&\approx \dfrac{1}{P}\left( \text{max }(s)-\bar{s} \right) . \end{aligned} \end{aligned}$$

This measure is large when the network is a star, i.e. the network contains one highly connected node.

Finally the variation of the strength of the nodes might be of interest. Heterogeneity equals the coefficient of variation of the strength distribution:

9 $${\text{Heterogeneity}}\,({\mathbf{M}}) = \frac{{\sqrt {{\text{ var }}(s)} }}{{\bar{s}}}.$$

These measures can be computed for a total network and for subnetworks or modules.

## Application to data

We will now analyze, describe and visualize the correlation structure of two metabolites datasets by using the described network approaches. The datasets are from an experiment aimed to study desiccation tolerance in germinated Arabidopsis seeds (Maia et al. 2014) and from an epidemiological cohort which studies the relationship between dietary, lifestyle, and genetic determinants and obesity and metabolic syndrome (DILGOM), which is a subset of the Finrisk 2007 survey (Inouye et al. 2010; Kettunen et al. 2012). The studies differ in the types of subjects (plants vs. human), the study designs [experimental design (completely randomized) vs. random sample of the population] and in sizes (27 vs. 419). For both studies about the same number of metabolites are available, namely 56 and 58 metabolites for the experiment and epidemiological studies, respectively.

### Experimental design

Desiccation tolerance (DT) is the ability of certain organisms to lose most of their cellular water content and become extremely dry and re-hydrate without the accumulation of lethal damage. DT is common in seeds of land plants. Such seeds acquire DT during development and become sensitive again to extreme dehydration around the point of visible germination. Yet, if confronted with suboptimal conditions, such as osmotic stress, germinated desiccation sensitive seeds are able to activate global changes in gene expression and metabolite composition to re-establish DT (Maia et al. 2011). Here, we are interested in the network structure of the metabolic phenotype of two Arabidopsis genotypes, a Wild type, Col-0, and an abscisic acid-insensitive (*aba2–1* mutant). Germinated seeds were subjected to a set of treatments including the application of osmotic stress by polyethylene glycol (PEG) and abscisic acid (ABA) to re-establish DT. In addition to the network structure of the observed metabolite concentrations also the network structure with regard to the relationship between the metabolites and the genotype (wild type and *aba2–1* mutant) will be studied.

In total 56 metabolites were measured for 15 samples of Arabidosis wildtype seeds and 12 samples of *aba2–1* mutant seeds. In Table 1, the number of samples for each combination of genotypes (Col-0, *aba2–1*) and treatment (control or no treatment), −2.5 MPa polyethylene glycol (PEG), 5 μM Abscisic acid (ABA) or both (PEG+ABA) are given.

**Table 1:** Experimental design for plant data

	No ABA		Yes ABA	
	No PEG	Yes PEG	No PEG	Yes PEG
Col-0	3	6	3	3
*aba2–1*	3	3	3	3

Cell counts denote the number of samples obtained per combination of treatment and Genotype

To obtain the genotypic part of the metabolites we fitted Eq. 2 to the data where $\mathbf {X}_m=\mathbf {G}$ represents the genotype; the four treatment levels were represented by two dummy variables denoting the administration of PEG and ABA. Thus, $\mathbf {X}^{(-m)}=(\mathbf {PEG},\mathbf {ABA})$. Specifically for the* p*th metabolite, the concentration associated with the seed’s genotype is given by:

10 $$\begin{aligned} \tilde{\mathbf {Y}}^{(p)}=\hat{\varvec{\beta }}_1^{(p)}\mathbf {G} +\hat{\varvec{\eta }}^{(p)}_{10}\mathbf {G}\circ \mathbf {PEG}+ \hat{\varvec{\eta }}^{(p)}_{01}\mathbf {G}\circ \mathbf {ABA}+ \hat{\varvec{\eta }}^{(p)}_{11}\mathbf {G}\circ \mathbf {PEG}\circ \mathbf {ABA}. \end{aligned}$$

Only significant variables were included in the models. Here we applied a hierarchical approach: if the highest order interaction was significant, all terms were included in the model. If the highest order interaction was not significant it was removed from the model and the significance of the consecutive highest order interaction terms was checked. Finally if the genetic effect was not significant, the metabolite was discarded from further analysis. 48 metabolites were used for the network analysis of genotype related metabolic variation.

Hereafter we will denote networks of metabolites containing the original metabolite values $\left( {{\mathbf{Y}}^{{(p)}} } \right)$ as ${\mathcal {N}}_O$ and networks containing the genotype related values $\left({\tilde{\mathbf {Y}}^{(p)}}\right)$ as ${\mathcal {N}}_G$.

#### Networks estimated by WGCNA

For estimation of the intensity matrix $\mathbf {W}$ we first tried to find the soft thresholding parameter $\gamma$ corresponding to a scale free topology. However it appeared that for reasonable powers ($\gamma$ being <12) both networks (${\mathcal {N}}_O$ and ${\mathcal {N}}_G$) were not scale-free. Therefore based on the sample size ($N=27$) and the number of metabolites ($P=54$ for ${\mathcal {N}}_O$ and $P=48$ for ${\mathcal {N}}_G$), $\gamma =5$ was chosen for suppressing low correlations due to noise (see Sect. 2.2.1).

**Fig. 2:**
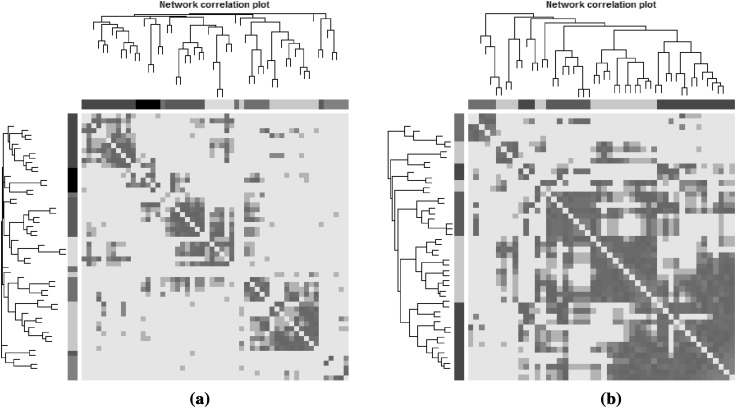
Heatmap plots for plant metabolites. Heatmaps were estimated for **a** ${\mathcal {N}}_O$ when the original metabolite values were used, and **b** ${\mathcal {N}}_G$ when using the metabolite information related to the Genotype.* Deep red colors* high values of absolute correlation between pairs of metabolites, while* lighter colors* correspond to weaker correlations. Dendrograms were obtained using hierarchical clustering while modules correspond to square blocks along the diagonal. Interconnected modules are color coded by using the color bands beneath the displayed dendrograms

In Fig. 2, the intensity matrices $\mathbf {W}_{{\mathcal {N}}_O}$ and $\mathbf {W}_{{\mathcal {N}}_G}$ are depicted together with their corresponding dendrograms obtained by average linkage hierarchical clustering. The visualization of the heatmaps reveals higher correlations (deeper red colors) between the genotype related metabolite values in (${\mathcal {N}}_G$) compared to the original metabolite values (${\mathcal {N}}_O$).

**Table 2:** Network statistics when using WGCNA (density, centralization, heterogeneity) for modules and network as whole in plant data

Module	Density	Centralization	Heterogeneity	Nr nodes	Color coded
${\mathcal {N}}_{O}$ ^a^					
Module 1^b^	0.33	0.20	0.33	6	Green
Module 2^c^	0.30	0.18	0.35	11	Turquoise
Module 3^d^	0.27	0.14	0.41	9	Brown
Module 4^e^	0.23	0.22	0.56	7	Yellow
Module 5^f^	0.20	0.12	0.31	11	Blue
Module 6^g^	0.19	0.13	0.41	5	Black
Module 7^h^	0.18	0.17	0.30	5	Red
Complete	0.06	0.04	0.45	54	
${\mathcal {N}}_G$ ^i^					
Module 8^j^	0.62	0.15	0.17	8	Brown
Module 9^k^	0.53	0.26	0.30	5	Yellow
Module 10^l^	0.42	0.17	0.31	17	Blue
Module 11^m^	0.38	0.19	0.33	18	Turquoise
Complete	0.21	0.16	0.44	48	

^a^Network using the original metabolite values

^b^Module 1: fructose, fructose-6-phosphate, glucose, glucose-6-phosphate, glyceric-acid, xylose

^c^Module 2: 2-aminoadipic-acid, glutamine, isoleucine, lysine, nicotinic-acid, phenylalanine, proline, pyroglutamic-acid, threonine, tyrosine, valine

^d^Module 3: 5-aminocarboxy-4,6-dihydroxypyrimidine, ascorbic-acid, glutamate, glycerol, hexonic-acid, monothylphosphate, phosphoric-acid, threonate, urea

^e^Module 4: aspartate, beta-alanine, citrate, ethanolamine, maltose, serine, tryptophan

^f^Module 5: alpha-ketoglutaric acid, allantoine, asparagine, fucose, galactinol, glycine, leucine, malate, raffinose , suberyl-glycine, succinic acid

^g^Module 6: alanine, methionine, myo-inositol, pentonic acid, sucrose

^h^Module 7: anhydroglucose, benzoic acid, fumarate, trans-sinapinic acid, xylofuranose

^i^Network using genotypic-related information

^j^Module 8: alanine, fructose, fructose-6-phosphate, fumarate, glucose-6-phosphate, threonate, trans-sinapinic acid, tyrosine

^k^Module 9: 2-aminoadipic-acid, anhydroglucose, benzoic-acid, maltose, xylofuranose

^l^Module 10: ascorbic-acid, aspartate, glucose, glutamine, glyceric-acid, isoleucine, leucine, lysine, raffinose, methionine, monomethylphosphate, phenylalanine, serine, threonine, tryptophan, valine, xylose

^m^Module 11: 5-aminocarboxy-4,6-dihydroxypyrimidine, allantoine, asparagine, citrate, galactinol, glycerol, glycine, hexonic acid, malate, myo-inosytol, pentonic acid, phosphoric acid, proline, pyroglutamic acid, suberyl-glycine, succinic acid, sucrose, urea

**Table 3:** Characterization of modules and network when estimating networks by GL and by using density, centralization and heterogeneity (edges’ stability has been used as weight) in plant data

Module	Density	Centralization	Heterogeneity	Nr nodes	Color coded
${\mathcal {N}}_O$ ^a^					
Module 1^b^	0.51	0.33	0.37	7	Brown
Module 2^c^	0.39	0.25	0.40	6	Yellow
Module 3^d^	0.23	0.19	0.48	10	Blue
Module 4^e^	0.22	0.19	0.46	10	Turquoise
Complete	0.05	0.07	0.71	54	
${\mathcal {N}}_G$ ^f^					
Module 5^g^	0.49	0.33	0.41	7	Yellow
Module 6^h^	0.37	0.23	0.31	9	Turquoise
Module 7^i^	0.29	0.33	0.48	8	Brown
Module 8^j^	0.25	0.21	0.44	9	Blue
Complete	0.05	0.07	0.77	48	

^a^Network using the original metabolite values

^b^Module 1: glutamine, isoleucine, lysine, pyroglutamic acid, threonine, tyrosine, valine

^c^Module 2: allantoine, galactinol, glycine, leucine, raffinose , succinic acid

^d^Module 3: 2-aminoadipic acid, alanine, aspartate, beta-alanine, citrate, methionine, phenylalanine, proline, perine, tryptophan

^e^Module 4: 5-aminocarboxy-4,6-dihydroxypyrimidine, ascorbic acid, fructose, fructose-6-phospate, glucose-6-phosphate, glyceric acid, glycerol, monomethylphosphate, pentonic acid, phosphoric acid, sucrose, threonate, urea

^f^Network using genotypic-related information

^g^Module 5: glutamine, glyceric acid, isoleucine, leucine, monomethylphosphate, valine, xylose

^h^Module 6: ascorbic-acid, aspartate, glycine, phenylalanine, proline, pyroglutamic acid, raffinose, serine, succinic acid

^i^Module 7: alanine, fructose-6-phospate, fumarate, glucose-6-phosphate, lysine, threonate, threonine, tyrosine

^j^Module 8: allantoine, asparagine, galactinol, glycerol, pentonic acid, phosphoric acid, suberyl-glycine, sucrose, urea

As seen in Table 2, the density and centralization of the complete networks are relatively low. The network ${\mathcal {N}}_G$ has a density of 0.21 and centralization of 0.16. The network ${\mathcal {N}}_O$ has a density of 0.06 and a centralization of 0.04. With regard to heterogeneity the two networks are similar (${\mathcal {N}}_G$ 0.44 and ${\mathcal {N}}_O$ 0.45). The two most dense modules of ${\mathcal {N}}_G$ have a moderate density of 0.62 and 0.53, but still small centralizations (0.15 and 0.26). The modules of ${\mathcal {N}}_O$ have small densities, namely smaller than 0.33.

**Fig. 3:**
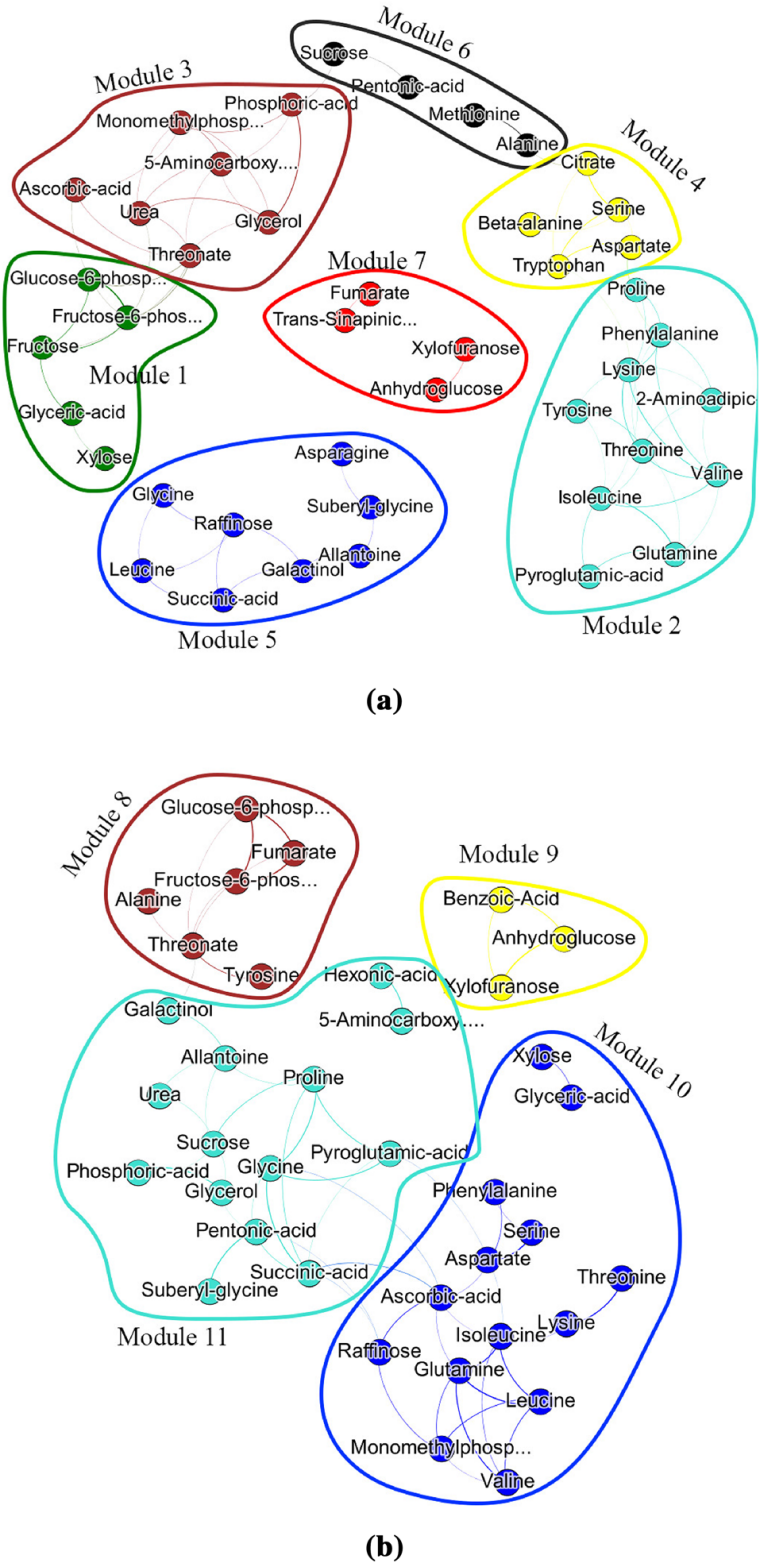
Sparse plant metabolite networks estimated by WGCNA with** a** the original metabolite values and **b** the Genotype related metabolite values. Modules have been color coded as indicated by column “Color Coded” in Table 2 in their corresponding network (${\mathcal {N}}_O$ or ${\mathcal {N}}_G$). Colors have been selected by the two-step dynamic hybrid algorithm implemented in the R-package WGCNA

In Fig. 3, the top $5\%$ of the strongest edges are visualized for the network ${\mathcal {N}}_O$ and ${\mathcal {N}}_G$. Here, a threshold of 0.36 for ${\mathcal {N}}_O$ and of 0.73 for ${\mathcal {N}}_G$ was used for keeping the top $5\%$ of the edges.

#### Networks estimated by GL

**Fig. 4:**
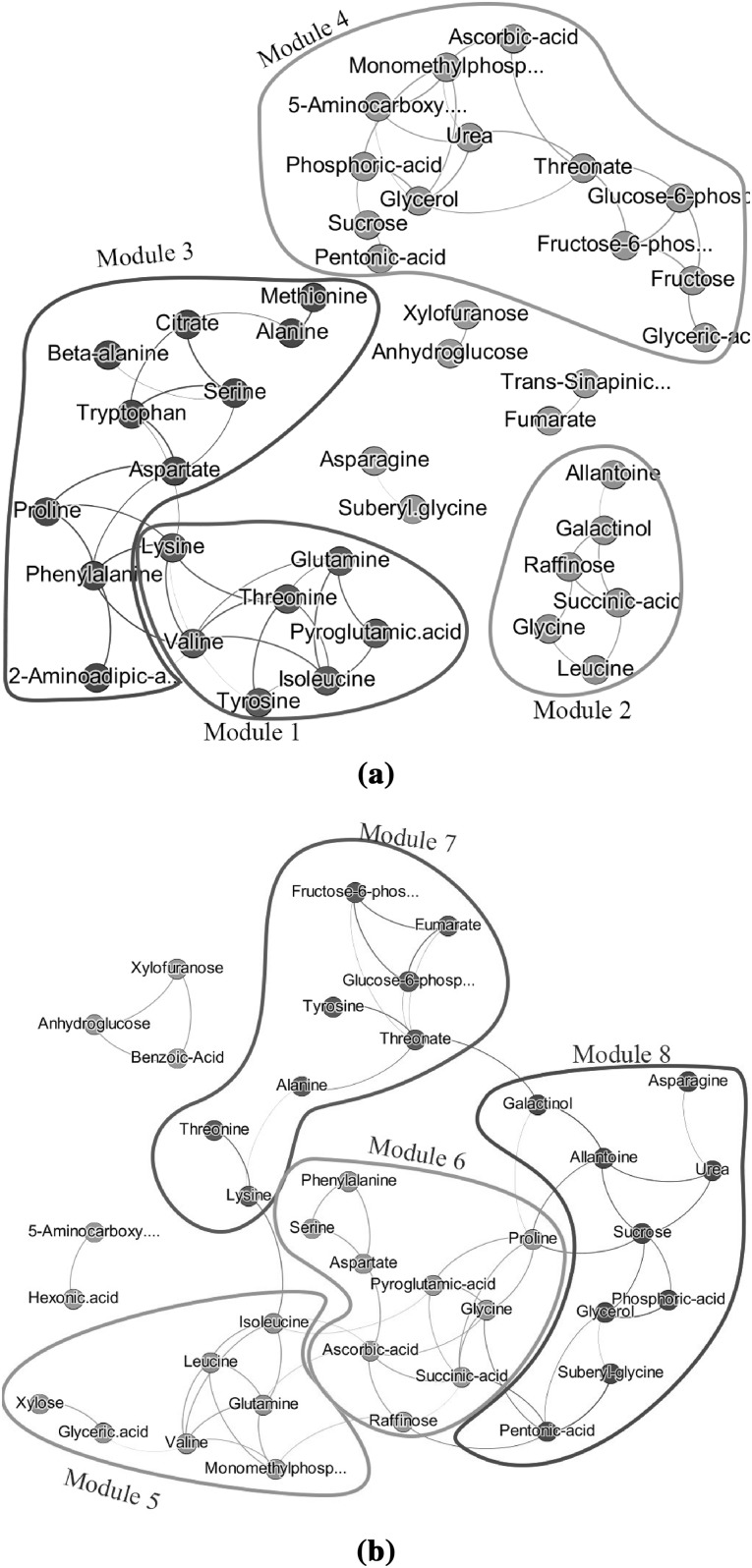
Estimated metabolite networks for plant data based on GL. In **a**, the estimated network is based on the original metabolite values, whereas in **b** Genotype related metabolite values have been used. Different modules have been color coded by the colors pinpointed in column ”Color Coded” of Table 3. The colors selection was guided by the two-step dynamic hybrid algorithm implemented in the R-package WGCNA

Next we consider the GL approach for estimation of networks. The regularization parameter $\lambda$ controlling the network’s sparsity was selected by using StARS (Liu et al. 2010). We randomly draw 100 subsamples of size 22 for estimating $\lambda$. A disagreement allowance of $5\%$, gave a $\lambda _{{\mathcal {N}}_O}$ of 0.82 and a $\lambda _{{\mathcal {N}}_G}$ of 0.94. In Fig. 4, the results are depicted. Here, the edges’ thickness and transparency denotes the edge’s stability i.e. frequency of edge occurrence in the 100 datasets. With regard to density, centralization and heterogeneity (Table 3) the two networks gave similar values. However, there was not much overlap between the identified modules of ${\mathcal {N}}_G$ and ${\mathcal {N}}_O$ obtained by the GL approach. Additionally, there does not seem to be much overlap between the modules obtained by different network estimation methods (WGCNA vs. GL).

*Top connected plant metabolites: * In Table 4, the top connected plant metabolites for the ${\mathcal {N}}_O$ and ${\mathcal {N}}_G$ networks which were estimated by WGCNA or GL are given. In general, the GL approach yielded higher degrees of the metabolites for the ${\mathcal {N}}_G$ than for the ${\mathcal {N}}_O$ network. This was not the case when the network was estimated by using WGCNA. Apart from small differences, the two methods (WGCNA and GL) appeared to give similar lists of top connected metabolites but the order was different. Additionally, the top connected metabolites of ${\mathcal {N}}_G$ were different from the ones of ${\mathcal {N}}_O$ in both WGCNA and GL cases. These results were also observed in Figs. 3 and 4.

**Table 4:** List of top connected plant metabolites for network estimation using WGCNA and GL

WGCNA			
${\mathcal {N}}_O$ ^a^		${\mathcal {N}}_G$ ^b^	
Metabolite	Degree	Metabolite	Degree
**Lysine**	8	*Threonate*	6
**Threonine**	7	***Succinic-acid***	6
*Threonate*	7	*Isoleucine*	6
*Valine*	6	***Ascorbic-acid***	6
**Phenylalanine**	6	***Sucrose***	5
*Isoleucine*	6	***Proline***	5
**Urea**	5	***Pentonic-acid***	5
*Monomethylophosphate*	5	***Glycine***	5
**Glycerol**	5	***Glutamine***	5
**Fructose-6-phosphate**	5	*Valine*	4
**5-Aminocarboxy-4,6-dihydroxypyrimidine**	5	***Pyroglutamic-acid***	4
**Tryptophan**	4	*Monomethylophosphate*	4
**Serine**	4	*Raffinose*	4
**Phosphoric-acid**	4	***Leucine***	4
*Raffinose*	4	***Allantoine***	4

GL			
${\mathcal {N}}_O^{\mathrm{a}}$		${\mathcal {N}}_G^{\mathrm{b}}$	
Metabolite	Degree	Metabolite	Degree
*Valine*	6	*Threonate*	6
**Threonine**	5	*Succinic-acid*	6
*Threonate*	5	*Proline*	6
**Phenylalanine**	5	*Isoleucine*	6
*Monomethylophosphate*	5	***Ascorbic-acid***	6
**Lysine**	5	*Valine*	5
*Isoleucine*	5	***Sucrose***	5
*Glycerol*	5	***Pentonic-acid***	5
**Urea**	4	***Glycine***	5
**Tryptophan**	4	*Glutamine*	5
**Serine**	4	***Pyroglutamic-acid***	4
*Glutamine*	4	*Monomethylophosphate*	4
**Aspartate**	4	***Raffinose***	4
*Succinic-acid*	3	***Leucine***	4
*Proline*	3	*Glycerol*	4

Results are displayed for the ${\mathcal {N}}_O$ and ${\mathcal {N}}_G$ networks. Italic denotes that the metabolite appears in both ${\mathcal {N}}_O$ and ${\mathcal {N}}_G$ networks. Bold is for metabolites that appear only in the list of ${\mathcal {N}}_O$ network and bolditalic for metabolites that appear only in the list of the ${\mathcal {N}}_G$ network

^a^ ${\mathcal {N}}_O$ network using the original metabolite values

^b^ ${\mathcal {N}}_G$ network using genotypic-related information

### Epidemiological study (DILGOM)

The other metabolomics dataset used has been measured in the epidemiological cohort DILGOM. A detailed description of the study and of the metabolomic dataset can be found in Inouye et al. (2010) and Kettunen et al. (2012). We excluded subjects who were diagnosed with diabetes, who received cholesterol medication, or who had outlying values for fasting glucose levels (>10 mmol/l). In addition only subjects with complete data were considered. After excluding these samples, we had 419 subjects (202 males and 217 females) aged between 25 and 74 years (median 53). The metabolomic data were measured by nuclear magnetic resonance (^1^H NMR) and comprise absolute quantitative measurements on 137 serum metabolites. Because of high correlation we removed 78 lipid particle subfractions and only the total lipid concentrations per particle size were used. Additionally, one more metabolite (FALEN) was excluded since its measurements were not completely trusted. Our final dataset consisted of $P=58$ metabolites: 25 lipoproteins, 13 lipids and fatty acids, 9 amino acids and 11 other small metabolites, e.g. involved in glycolysis. We adjusted all the metabolites for diastolic blood pressure (DBP) and blood pressure medication (BPM) (binary) by linear regression. In the rest of this section we will denote these adjusted metabolites as metabolites. We are interested in the network of metabolite concentrations and in the part of the metabolite concentrations related to BMI.

Since the metabolites and BMI depend on age and sex, these variables were included in model 2 as $\left( {{\mathbf{X}}_{1} ,{\mathbf{X}}_{2} } \right)$. The continuous BMI values were categorized into three equally sized classes (1st thirtile = 24.38, 2nd thirtile = 27.56). Thus in Eq. 2, the total number of covariables is $m=3$ and the variable $\mathbf X _3$ represents the indicator variables for the three BMI categories. The model 2 includes the main effects, first and second order interactions. Specifically for the *p*th metabolite, the following equation was used,

11 $$\begin{aligned} \tilde{\mathbf {Y}}^{(p)}=\hat{\varvec{\beta }}_1^{(p)}{\mathbf{BMI }}+ \hat{\varvec{\eta }}^{(p)}_{10}{\mathbf{BMI }}\circ {\mathbf{Age }}+\hat{\varvec{\eta }}^{(p)}_{01}{\mathbf{BMI }}\circ {\mathbf{Sex }}+\hat{\varvec{\eta }}^{(p)}_{11}{\mathbf{BMI }}\circ {\mathbf{Age }}\circ {\mathbf{Sex }}. \end{aligned}$$

Analogously to the plant application, networks of metabolites containing the original metabolite values $\left( {{\mathbf{Y}}^{{(p)}} } \right)$ are denoted by ${\mathcal {N}}_O$, and networks of metabolites containing the relevant information on BMI $\left({\tilde{\mathbf {Y}}^{(p)}} \right)$ by ${\mathcal {N}}_B$.

#### Networks estimated by WGCNA

**Fig. 5:**
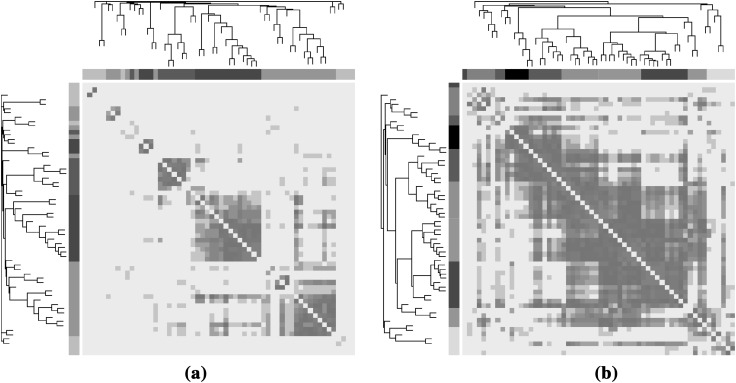
Correlation matrix plot. The plots were generated for **a** ${\mathcal {N}}_O$ using the original metabolite values, and **b** ${\mathcal {N}}_B$ using BMI information. Dendrograms were obtained by ordering metabolites using hierarchical clustering. Modules of interconnected metabolites correspond to* square blocks along the diagonal*, while* deep red colors* denote strong correlations (on absolute value). Metabolites belonging in the same module are color coded by the colors (coming from the two-step dynamic hybrid algorithm) that are indicated by the color band below each dendrogram

For estimation of the intensity matrix, we first tried to determine a soft-thresholding parameter $\gamma$ for which the network had a scale free topology. For both networks (the original and BMI related metabolite values), the scale-free topology did not hold for reasonable powers, i.e. $\gamma$ <12. Therefore, $\gamma =3$ was chosen based on the sample size ($n=419$) and the number of metabolites ($P=58$) as described in Sect. 2.2.1. The absolute values of Pearson’s correlation coefficients are visualized in Fig. 5; darker red colors represent strong correlations, and lighter colors weaker correlations. The heatmap of ${\mathcal {N}}_B$ shows several larger blocks of highly correlated metabolites while the heatmap of ${\mathcal {N}}_O$ shows several small and distinct clusters.

**Fig. 6:**
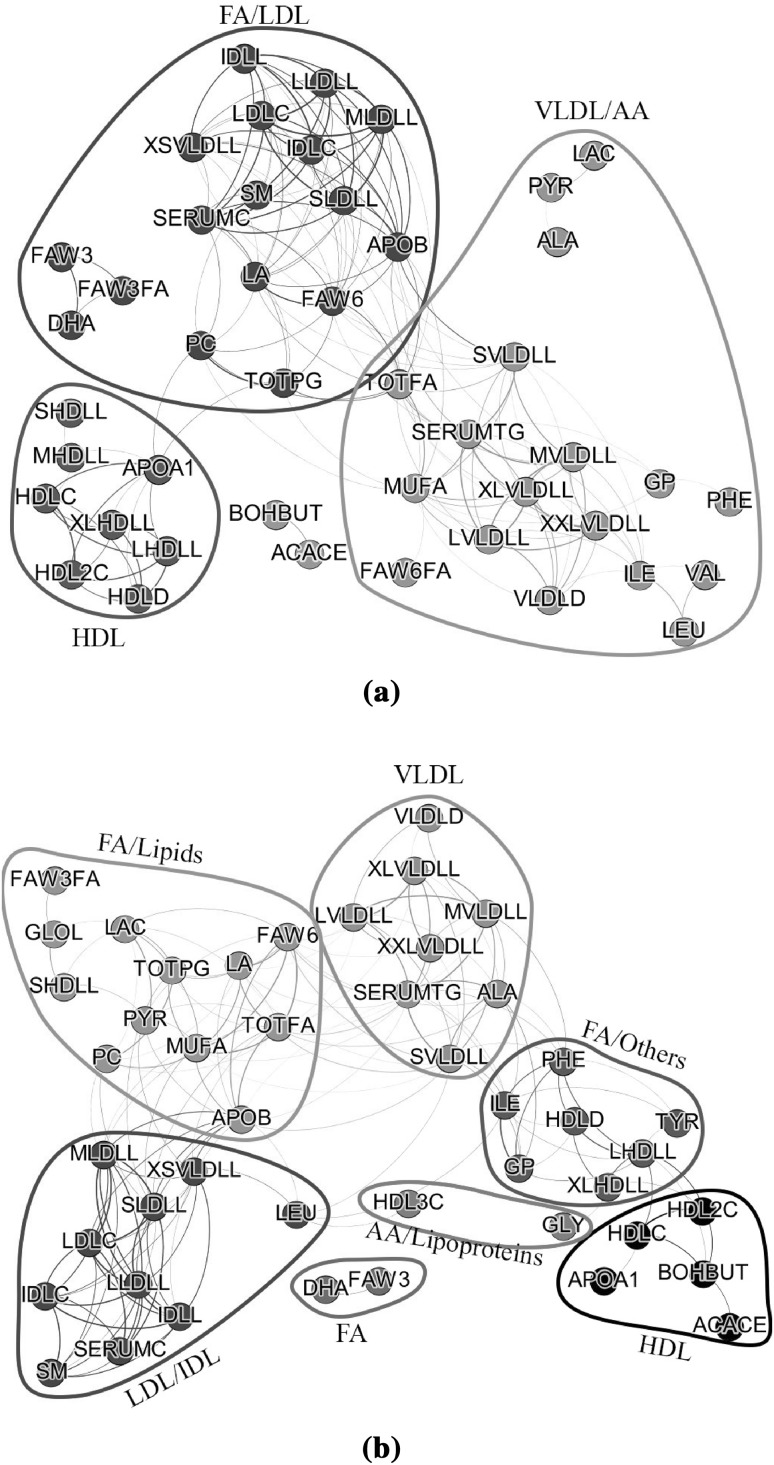
Estimated sparse metabolite networks in humans based on WGCNA. In **a**, the network is based on the original metabolite values. In **b** the BMI related metabolite network is displayed. Metabolites have been *colored* by the *color* of the module they belong to. Information on the *color* of each module can be found in Table 5. *Colors* were selected based on the two-step dynamic hybrid algorithm implemented in the R-package WGCNA

For visualizing and depicting edges in both networks we used the following thresholds: 0.22 for ${\mathcal {N}}_O$ and 0.66 for ${\mathcal {N}}_B$. These thresholds correspond to the top $10\%$ of the edges (Fig. 6). For identifying interconnected modules, we applied average linkage hierarchical clustering and obtained dendograms. Modules were defined as branches of the dendogram and were identified using the two-step dynamic hybrid algorithm. The descriptives of the two networks are given in Table 5. The complete network ${\mathcal {N}}_B$ has a larger density (0.23 vs. 0.07) and centralization (0.17 vs. 0.11) and a lower heterogeneity value (0.50 vs. 0.76) than the complete network ${\mathcal {N}}_O$. The first three modules of ${\mathcal {N}}_B$ are more dense than the modules of $N_O$ (0.63–0.77 vs. 0.15–0.32). The centralization and the heterogeneity of the modules of $N_G$ are smaller than of the modules of $N_O$. In both cases, centralization exhibits relatively low values denoting that there is not a dominant metabolite in each of the modules.

**Table 5:** Characterization of modules and network in humans with WGCNA using density, centralization and heterogeneity

Module	Density	Centralization	Heterogeneity	Nr nodes	Color coded
${\mathcal {N}}_O$ ^a^					
FA/LDL^b^	0.32	0.19	0.45	17	Blue
HDL^c^	0.31	0.23	0.55	9	Brown
VLDL/AA^d^	0.15	0.19	0.72	22	Turquoise
Complete	0.07	0.11	0.76	58	
${\mathcal {N}}_B$ ^b^					
VLDL^e^	0.77	0.11	0.11	8	Yellow
HDL^g^	0.67	0.15	0.15	5	Black
LDL/IDL^h^	0.63	0.18	0.35	11	Blue
FA/lipids^i^	0.59	0.16	0.19	13	Turquoise
FA/others^j^	0.54	0.15	0.27	9	Brown
FA^k^	0.20	0.20	0.42	6	Green
AA/lipoproteins^l^	0.19	0.16	0.38	6	Red
Complete	0.23	0.17	0.50	58	

^a^Network using the original metabolite values

^b^FA/LDL: APOB, DHA, FAW3, FAW3FA, FAW6,IDLC, IDLL, LA, LDLC, LLDLL, MLDLL, PC, SERUMC, SLDLL, SM, TOTPG, XSVLDLL

^c^HDL: ALB, APOA1, HDL2C, HDLC, LHDLL, MHDLL, SHDLL, XLHDLL

^d^VLDL/AA: ALA, FAW6FA, GLC, GP, HDL3C, ILE, LAC, LDLD, LEU, LVLDLL, MUFA, MVLDLL, PHE, PYR, SERUMTG, SVLDLL, TOTFA, TYR, VAL, VLDLD, XLVLDLL, XXLVLDLL

^e^Network using BMI-related information

^f^VLDL: ALA, LVLDLL, MVLDLL, SERUMTG, SVLDLL, VLDLD, XLVLDLL, XXLVLDLL

^g^HDL: ACACE, APOA1, BOHBUT, HDL2C, HDLC

^h^IDL/LDL: ACE, IDLC, IDLL, LDLC, LEU, LLDLL, MLDLL, SERUMC, SLDLL, SM, XSVLDLL

^i^FA/lipids: APOB, FAW3FA, FAW6, GLOL, LA, LAC, LDLD, MUFA, PC, PYR, SHDLL, TOTFA, TOTPG

^j^FA/others: GLC, GP, HDLD, ILE, LHDLL, PHE, TYR, VAL, XLHDLL

^k^FA: CIT, DHA, FAW3, FAW6FA, GLN, MHDLL

^l^AA/lipoproteins: ALB, CREA, GLY, HDL3C, HIS, UREA

**Table 6:** Characterization of modules and network in GL using density, centralization and heterogeneity (edges stability has been used as weight) in humans

Module	Density	Centralization	Heterogeneity	Nr nodes	Color coded
${\mathcal {N}}_O$ ^a^					
LDL/IDL^b^	0.71	0.31	0.34	9	Blue
FA^c^	0.65	0.45	0.28	6	Yellow
BCAA/VLDL^d^	0.56	0.23	0.38	9	Turquoise
HDL^e^	0.45	0.27	0.45	6	Brown
Complete	0.07	0.17	0.98	58	
${\mathcal {N}}_B$ ^f^					
LDL/IDL^g^	0.79	0.27	0.24	9	Blue
VLDL^h^	0.61	0.30	0.35	8	Yellow
FA^i^	0.44	0.56	0.45	9	Brown
Lipids/HDL^j^	0.30	0.31	0.42	10	Turquoise
Complete	0.05	0.11	0.98	58	

^a^Network using the original metabolite values

^b^LDL/IDL: IDLC, IDLL, LDLC, LLDLL, MLDLL, SERUMC, SLDLL, SM, XSVLDLL

^c^FA: APOB, FAW6, LA, MUFA, SVLDLL, TOTFA

^d^BCAA/VLDL: ILE, LEU, LVLDLL, MVLDLL, SERUMTG, VAL, VLDLD, XLVLDLL, XXLVLDLL

^e^HDL: APOA1, HDL2C, HDLC, HDLD, LHDLL, MHDLL, PC, TOTPG, XLHDLL

^f^Network using BMI-related information

^g^LDL/IDL: IDLC, IDLL, LDLC, LLDLL, MLDLL, SERUMC, SLDLL, SM, XSVLDLL

^h^VLDL: ALA, LVLDLL, MVLDLL, SERUMTG, SVLDLL, VLDLD, XLVLDLL, XXLVLDLL

^i^FA: APOB, FAW6, LA, LAC, MUFA, PC, PYR, TOTFA, TOTPG

^j^Lipids/HDL: ACACE, BOHBUT, GP, HDL2C, HDLC, HDLD, ILE, LHDLL, PHE, XLHDLL

#### Networks estimated by GL

**Fig. 7:**
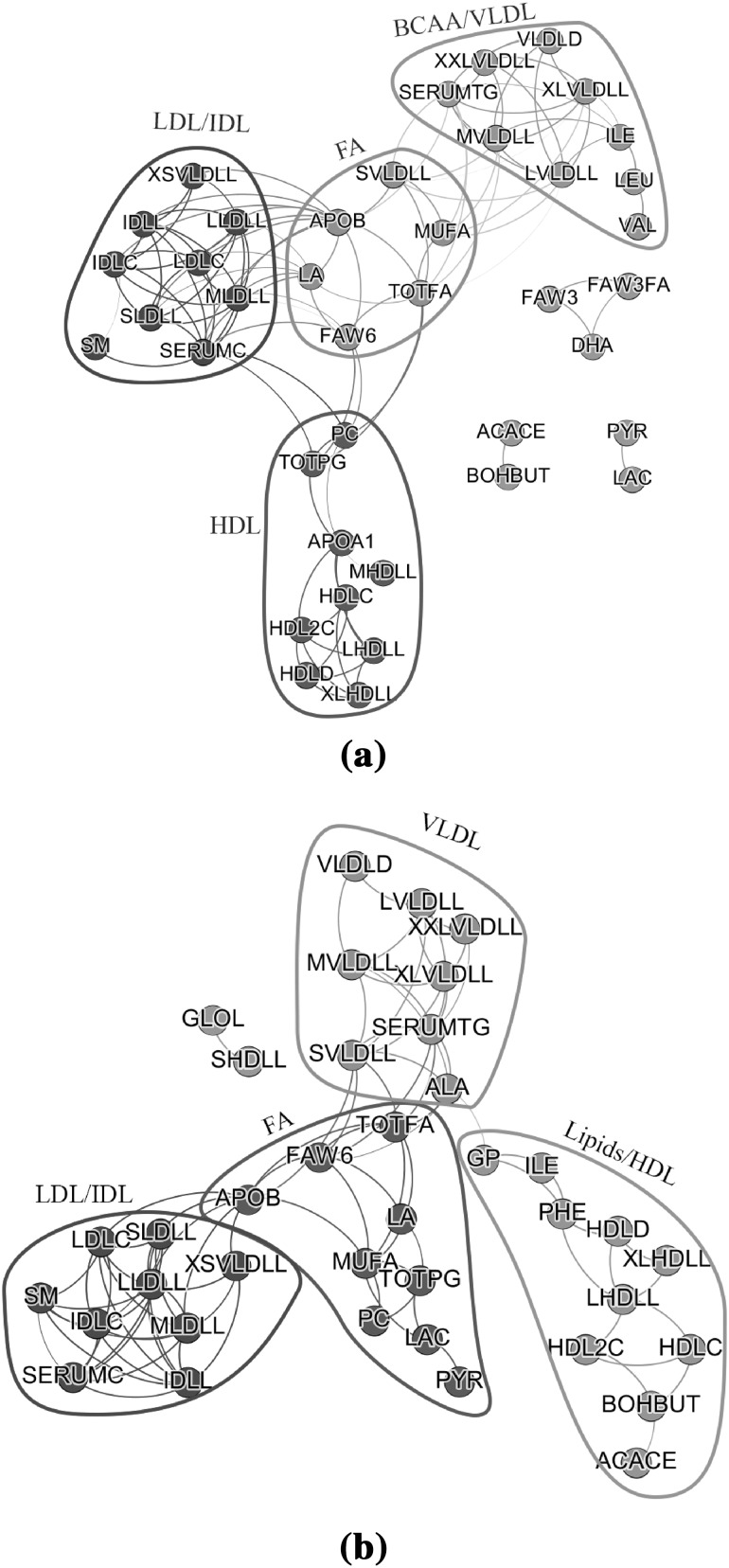
Sparse metabolite networks estimated by GL. The networks were constructed **a** using the original metabolite values, and **b** using the metabolite values driven by variation originating from BMI. Metabolites were *colored* based on the module they belong to. Information on the membership of each metabolite and on the *color* they have been *colored* can be found in Table 6

Next we considered the GL approach. The penalty parameter $\lambda$ was selected by using StARS (Liu et al. 2010). We used a size for the subsamples of 205 (almost $50\%$ of the total sample size) and a disagreement allowance of $2\%$ across the networks. After subsampling 100 times, we obtained a $\lambda _{{\mathcal {N}}_O}$ of 0.68 and $\lambda _{{\mathcal {N}}_B}$ of 0.91. Fig. 7 depicts the results. Here the edge thickness represent the stability of the estimated edges. The evaluation measures are also calculated based on stability (Table 6). The density, centralization, and heterogeneity was very similar for both networks. The first modules of each network contained exactly the same metabolites and had also very similar properties. Interestingly the second dense module of ${\mathcal {N}}_G$ (VLDL) and third of the ${\mathcal {N}}_O$ (BCAA/VLDL) had a large overlap with regard to VLDL particles. Note that VLDL particles were also identified by the WGCNA approach as a module. In Fig. 1 metabolite concentrations stratified by BMI and Sex are depicted for the VLDL module. It appeared that all these eight metabolites had a high value in obese men. For females the values were much lower for these metabolites except for alanine. Note that alanine is also on the border of the cluster (Figs. 6b,  7b) and shares connections with metabolites from other modules. Finally, the third densest module in ${\mathcal {N}}_B$ (FA) identified by GL exhibits a relatively high centralization. This denotes that some metabolite(s) had higher than average degree (hub metabolite(s)). The metabolite with the highest strength (6.99) and degree (7) as measured by the stability in GL within the module is monounsaturated fatty acids (MUFA). MUFA being connected to all metabolites in the FA module (only pyruvate is not a direct connection) indicates that it might have the most representative metabolite profile among the rest. LLDLL (total lipids of large LDL particles), with eight connections and strength 7.98 is the metabolite with the most connections within the Lipoproteins module. In the VLDL module, LVLDLL has the most connections (six connections) with strength of 5.98.

*Top connected metabolites* In Table 7 the top 15 metabolites are given for combinations of the two approaches (WGCNA and GL) and two types of correlation structures (metabolites and BMI specific metabolites). The degree *k* of the metabolites was higher for WGCNA than for GL. The same conclusion holds when comparing Figs. 6b,  7b.

**Table 7:** List of top connected plant metabolites for network estimation using WGCNA and GL

WGCNA			
${\mathcal {N}}_O$ ^a^		${\mathcal {N}}_B$ ^b^	
Metabolite	Degree	Metabolite	Degree
*TOTFA*	16	*TOTFA*	17
*FAW6*	16	*SVLDLL*	15
*APOB*	15	*APOB*	15
*SVLDLL*	14	*SERUMTG*	14
**SERUMC**	14	*SLDLL*	14
**LA**	14	*FAW6*	14
*MUFA*	14	*MLDLL*	12
*XSVLDLL*	12	*MUFA*	12
*MLDLL*	12	***ALA***	11
*SLDLL*	12	***LVLDLL***	10
*SERUMTG*	12	*MVLDLL*	10
*MVLDLL*	11	*XSVLDLL*	10
*IDLL*	11	***XLVLDLL***	9
**LLDLL**	11	*IDLL*	9
**IDLC**	11	***ILE***	9

GL			
${\mathcal {N}}_O$ ^a^		${\mathcal {N}}_B$ ^b^	
Metabolite	Degree	Metabolite	Degree
*APOB*	13	*LLDLL*	9
*SERUMC*	12	*MLDLL*	9
*SERUMTG*	10	*APOB*	9
*TOTFA*	10	***SVLDLL***	8
*LVLDLL*	9	*IDLL*	8
*MVLDLL*	9	*LDLC*	8
*LLDLL*	9	*SERUMTG*	8
*MLDLL*	9	*SLDLL*	7
*SLDLL*	9	*IDLC*	7
*IDLC*	9	*SERUMC*	7
**XLVLDLL**	8	*FAW6*	7
*IDLL*	8	*TOTFA*	7
*LDLC*	8	***MUFA***	7
*FAW6*	8	*LVLDLL*	6
**LA**	8	*MVLDLL*	5

Results are displayed for the ${\mathcal {N}}_O$ and ${\mathcal {N}}_G$ networks. Italic denotes that the metabolite appears in both ${\mathcal {N}}_O$ and ${\mathcal {N}}_G$ networks. Bold is for metabolites that appear only in the list of ${\mathcal {N}}_O$ network and Bolditalic for metabolites that appear only in the list of the ${\mathcal {N}}_G$ network

^a^Network using the original metabolite values

^b^Network using BMI-related information

## Discussion

In this paper we studied and visualized the correlation structure of two datasets of metabolomics coming from two different studies. The studies differ in types of subjects, study designs and sizes. We considered two methodological approaches to estimate the networks: namely WGCNA based on correlation and GL based on partial correlation. The methods were applied to the metabolite values and to parts of the metabolite values which incorporate the effect of a covariable of interest, e.g. genotype or BMI. We compared the obtained networks in terms of density, centralization and heterogeneity of the relationships between the nodes.

The density of the networks based on the covariable specific part of the metabolites had larger or similar values than the density of the networks based on the metabolites itself. Interestingly, for the metabolites measured in the epidemiological study, a number of lipoproteins (i.e. IDLC, IDLL, LDLC, LLDLL, SERUMC, SLDLL, SM, XSVLDLL) was clustered together when keeping the original or the BMI-specific metabolite values in both network estimation methods. In addition, for the BMI specific part both approaches identified exactly the same module consisting mainly of VLDL particles with a density of 0.77 using Pearson correlation, and a density of 0.61 using stability in the GL approach. This module is characterized by high values for obese men. The relationship between BMI and VLDL concentrations is known. In several studies it has been found that obese people have elevated VLDL concentrations (almost by 50%; Magkos et al. 2008) compared to lean individuals (Mittendorfer et al. 2003; Chan et al. 2004; Goff et al. 2005; Magkos et al. 2008; Magkos and Mittendorfer 2009). This can be attributed to the hepatic overproduction of VLDL particles (Chan et al. 2004; Ooi et al. 2005) which characterizes obesity. Hepatic overproduction of VLDL particles is also stimulated by atherogenic dislipidemia (which is commonly present in obese people) and promoted by increased liver fat (Grundy 2004; Klop et al. 2013), also common in obese people. Finally, abdominal fat (known as visceral adipose tissue) and BMI have been found to be positively associated to VLDL particle concentrations and size suggesting again the association between obesity and high levels of VLDL (Sam et al. 2008).

The results for the data from the Arabidopsis desiccation tolerance experiments were harder to interpret. In general, the densities and centralizations of the networks were smaller. This might be the result of the small sample size. Indeed the $\gamma$ parameter to shrink small values in the WGCNA approach is larger for the experimental than for the epidemiological design (five and three respectively). However for the GL approach there was less difference in the shrinkage, namely the shrinkage parameter was 0.68 and 0.91 for $N_O$ and $N_B$ in the epidemiology study and 0.82 and 0.94 for $N_O$ and $N_G$ in the experimental design. While in the epidemiological study the network specific to BMI provided interesting results, this was less the case for the desiccation tolerance data. One reason may be that the main effect of the genotype on the metabolite variation was smaller than the main effect of the treatment. We presented a treatment corrected metabolic network reconstrution in which the wild type and mutant type genotypes are compared across treatments. This is a sensible analysis in plant genetics (Eeuwijk et al. 2010). However, from a seed physiological perspective an analysis correcting for genotype and comparing the treatments across the two genotypes may be more interesting. We will present the results of such an analysis elsewhere.

WGCNA and GL are complementary approaches for metabolite inference since the former recovers meaningful modules and the latter recovers meaningful edges, e.g. for the DILGOM study MUFA is the dominant metabolite in the FA module when GL is used. WGCNA is based on the correlation structure, and the obtained results are therefore straightforward to interpret. To reduce the noise in the data, a soft threshold is often applied to sufficiently shrink small correlations to zero. WGCNA allows detection of modules with high density. GL is based on Gaussian graphical models, in which the conditional independence is inferred by the zero entries in the precision matrix. In order to induce sparsity to the precision matrix and to estimate stable sets of edges with a low false discovery rate, a stability selection approach, StARS, was applied. This can lead to detection of modules with high centralization. For both approaches modules are selected by constructing a dendogram based on average linkage and cutting the branches.

In both cases, WGCNA and GL, the user has to choose specific tuning parameters: the soft threshold in WGCNA and the disagreement allowance and the number of the subsamples for GL with StARS. For a large dataset, StARS might not be needed and the penalty parameter might be chosen based on cross validation making the procedure to be data driven. The impact of the tuning parameters in WGCNA and GL is high; so data driven methods for selecting them would be ideal.

For clear visualization of the constructed networks using WGCNA, arbitrary thresholds were applied to depict the top $5\%$ of the edges in the experimental design and $10\%$ in the human data. These numbers were selected together with plant biologists and epidemiologists so that a set of meaningful edges was recovered by the data. The disagreement allowance parameter in GL was set to $5\%$ in the experimental design and $2\%$ in the epidemiological data for the same reason.

Apart from the two considered methods for estimating networks of metabolites, other well established methods have been used in practice based on conditional independence using regression techniques, ridge regularization and partial correlation. First, a simple approach for estimating sparse networks using regression techniques is by estimating the edge set for each variable by fitting a Lasso model to each variable using the remaining variables as predictors (Meinshausen and Bühlmann 2006). The non-zero Lasso coefficients identify the adjacent nodes to which each variable is connected to. The shortcoming of this method compared to GL which estimates the network structure simultaneously, is that an edge between two nodes (*p* and *q*) might be estimated from *p* to *q* but not vice versa. For overcoming this, an AND or an OR rule can be applied. This method asymptotically estimates the set of non zero elements of the precision matrix, but it does not yield the maximum likelihood estimator (Banerjee et al. 2008). In contrast to Lasso penalty in GL which estimates entries in the precision matrix (and subsequently in the adjacency matrix) exactly equal to zero, a ridge penalty can also be used in the penalized log likelihood (Ha and Sun 2014). The elements in the precision matrix will shrink, but will not be exactly zero unless a threshold is chosen for having exactly zeros (Efron 2004). In this case, GL seems to be advantageous due to the sparsity that is preferred for interpreting the results. Also, by applying a threshold in the ridge-based method, a second parameter should be estimated on top of the ridge penalty $\lambda _{\text{ ridge }}$. This usually involves a two-dimensional grid search approach that can be time-consuming and the optimization problem might not be convex as well. Finally, the edges of the network can be chosen by estimating Pearson’s partial correlation coefficient for any given pair of variables directly. Partial correlation eliminates edges that appear from indirect effects which is a desired characteristic (Krumsiek et al. 2011). The shortcoming of the method is that it is not applicable in the high dimensional setting. In this case, partial correlation cannot be estimated by either using the linear regression, or the matrix inversion methods. Using a recursive formula is also not possible and is computationally expensive. Therefore, here we used WGCNA which is a commonly used approach and is based on observed correlations which are easy to interpret. GL is also used since it has all desirable features (sparsity, computational speed, can be used in high-dimensional setting) that the other methods fall short of.

For identifying and selecting modules in this paper, the two-step dynamic hybrid algorithm was used with 1−*w*_*ij*_ as a dissimilarity measure in WGCNA and one minus stability of the edges (number of edges occurrences from the subsampling scheme) in GL. Another popular clustering method is the modularity optimization. Dividing a network in modules so that the modularity is optimal, will result in many edges within the modules and few edges between modules (Newman and Girvan 2004). Module identification by using modularity optimization is a known data-driven approach, which is used without specifying any arbitrary parameters. Here the dynamic hybrid algorithm was used instead, because while it takes the modular structure into account (through the dendrogram), it additionally does not have a constant cut-off height, so is able to identify nested clusters by using the cluster shape.

Some issues still need to be addressed. For example network estimation methods should thoroughly be investigated on the sample size sensitivity (large vs. small sample sizes) and in changes on tuning parameters (soft-threshold in WGCNA, disagreement allowance in GL). Further work for repeated measurements of the metabolites over time should be considered.

The regression framework to study specific parts of the metabolite concentrations worked well for the epidemiological dataset. We recovered sets of metabolites that are associated to a categorical covariable of interest in the same way. By using a network approach coupled with a module identification method, sets of metabolites which regulate the covariable of interest in the same way can be detected.
